# Right ventricular dysfunction in a hypertensive population stratified by patterns of left ventricular geometry

**DOI:** 10.5830/CVJA-2012-014

**Published:** 2012-10

**Authors:** Kamilu M Karaye, Hadiza Sai’du, Mohammed N Shehu

**Affiliations:** Department of Medicine, Bayero University, Kano, Nigeria; Aminu Kano Teaching Hospital, Kano, Nigeria; Aminu Kano Teaching Hospital, Kano, Nigeria; Aminu Kano Teaching Hospital, Kano, Nigeria

**Keywords:** hypertension, RV dysfunction, LV geometry, Nigeria

## Abstract

**Introduction:**

The aim of this study was to assess the prevalence, determinants and correlates of right ventricular (RV) systolic and diastolic dysfunction (RVSD and RVDD, respectively) in hypertensives, stratified by left ventricular (LV) geometric patterns.

**Methods:**

The study was carried out in Aminu Kano Teaching Hospital in Kano, Nigeria, and was cross-sectional in design. Hypertensive subjects referred for echocardiography were consecutively recruited after satisfying the inclusion criteria. RVSD was defined as either tricuspid annular plane systolic excursion (TAPSE) of < 16 mm, or peak velocity of the systolic wave (S_m_) in tissue Doppler imaging (TDI) of the RV lateral tricuspid annulus of < 10 cm/s, or both. RVDD was defined as the ratio of < 1.0 of the peak velocities of the early (E_m_) to late (A_m_) diastolic waves in the TDI of the RV lateral tricuspid annulus. Subjects with normal LV geometry (NG) served as controls, and were compared with those who had eccentric (EH) or concentric (CH) LV hypertrophy or concentric LV remodelling.

**Results:**

A total of 128 subjects were recruited. Overall, the prevalence of RVDD almost doubled that of RVSD in the studied subjects (61.72 vs 32.03%, respectively). Subjects with EH had the highest prevalence of RVSD (52.63%), while those with CH had the lowest prevalence (20.69%) (*p* < 0.01). By contrast, the prevalence of RVDD was high across the four groups without significant statistical difference; as high as 68.52% in subjects with NG and as low as 42.86% in those with CR. LVEF was the only independent determinant of RVSD after controlling for confounding variables, while age was the only determinant of RVDD. Likewise, age was the only correlate for E_m_:A_m_ ratio, while the best correlate for both TAPSE and S_m_ was LVEF.

**Conclusion:**

The study has revealed that about two-thirds of the hypertensives had RVDD while about one-third had RVSD. Subjects with EH had the highest prevalence of RVSD, while RVDD was common across all the groups. LVEF and age were the only independent determinants of RVSD and RVDD, respectively.

## Abstract

Right ventricular (RV) systolic and diastolic functions have repeatedly been studied in hypertensive subjects.^1^,^2^ Abnormal RV function has been found to be an independent, poor prognostic factor in subjects with heart failure (HF) of various aetiologies, including hypertension, and is associated with increased morbidity and mortality.^3^

Meluzin *et al.*^3^ assessed the prognostic power of RV systolic and diastolic functional parameters derived from Doppler tissue imaging (DTI) of tricuspid annular motion, and whether their combination might improve the risk stratification of patients with heart failure. They found that the combination of RV systolic and diastolic functional parameters represents a very powerful tool for risk stratification of patients with symptomatic heart failure.^3^

Abnormal LV geometry is also common in hypertensive subjects.^4^ Consideration of the level of LV mass and the LV wall thickness/chamber radius ratio [relative wall thickness (RWT)] has identified four different geometric patterns of LV adaptation to hypertension.^5^ These are concentric LV hypertrophy (CH) (increased mass and relative wall thickness), eccentric hypertrophy (EH) (increased mass, normal relative wall thickness), concentric remodelling (CR) (increased relative wall thickness with normal mass) and normal LV geometry (NR) (see Fig. 1).^5^

**Fig. 1.**
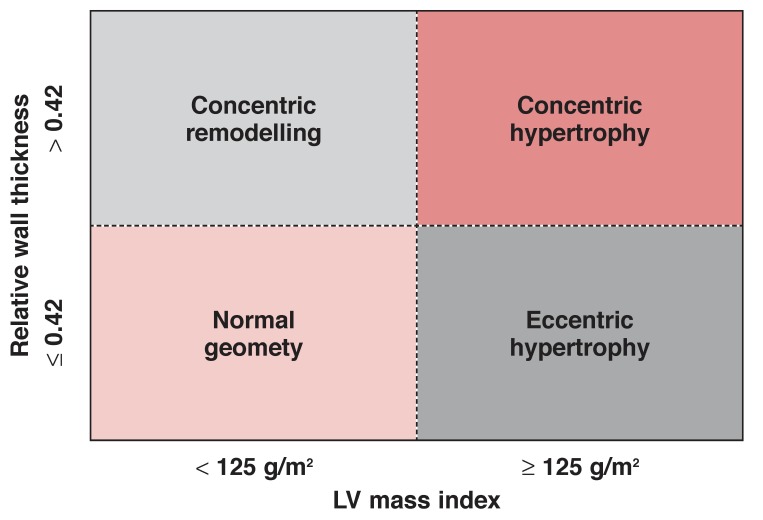
Determination of LV geometric patterns in subjects with hypertension.

Concentric hypertrophy is associated with especially high arterial pressure while eccentric hypertrophy is associated with obesity and elevated volume load.^5^ A long-term follow-up study has revealed that those with CH had the highest rates of all-cause mortality and cardiovascular morbid events, while patients with EH or CR had rates of morbidity that fell between those of patients with CH and the low-risk group with normal LV geometry.^6^

Although studies have shown that assessing the right and left ventricles are important in prognostication, and that hypertensive LV geometric patterns are different from each other in several respects, as mentioned above,^1^-^6^ it has not previously been well described whether RV function in subjects with the various LV geometric patterns are also different. The aims of the present study were therefore to assess the prevalence, determinants and correlates of RV systolic and diastolic dysfunction (RVSD and RVDD, respectively) in a hypertensive population, grouped according to the various LV geometric patterns. It is hoped that this information would further characterise the structure and function of both the right and left ventricles in hypertensive subjects.

## Methods

The study was carried out in the echocardiography laboratory of Aminu Kano Teaching Hospital in Kano, north-western Nigeria. The Research Ethics Committee of the Hospital reviewed and approved the study protocol, which conformed to the ethical guidelines of the Declaration of Helsinki, on the principles for medical research involving human subjects.^7^

The study was cross-sectional in design. Hypertensive subjects referred for echocardiography to Aminu Kano Teaching Hospital, Kano, Nigeria, were recruited consecutively from October 2009 to April 2010, after obtaining informed consent. Minimum sample size was estimated at 94 subjects using a validated formula,^8^ applying a prevalence of hypertensive heart disease (HHD) in Kano of 56.7% (among patients referred for echocardiography),^9^ and a sample error of 10%.

Transthoracic echocardiography was performed by the authors using the Aloka Cardiac Ultrasound System (model SSD 4000 PHD), and the procedures were carried out according to the recommendations of the American Society of Echocardiography.^10^ Left ventricular ejection fraction (LVEF) was calculated using Teicholz’s M-mode formula while LV mass index (LVMI) was calculated using Devereux’s formula.^11^,^12^ Patients were examined in the left lateral decubitus position.

Tricuspid annular plane systolic excursion (TAPSE) was recorded from the apical four-chamber view with the M-mode cursor positioned at the free-wall angle of the tricuspid valve annulus.^13^ Right ventricular long-axis excursion amplitude (i.e. TAPSE) was taken from end-systole to end-diastole.^13^ Tracings for TAPSE and TDI of the RV lateral tricuspid annulus were obtained from the apical approach during held end-expiration. Care was taken to align M-mode or TDI beam along the direction of tricuspid annulus motion. TDI sample volume was positioned 10 mm from the insertion site of the tricuspid leaflets or 10 mm away within the right ventricle lateral wall and adjusted to cover the longitudinal excursion of the tricuspid annulus in both systole and diastole.^14^

All the recruited subjects were hypertensive on treatment and in sinus rhythm. Subjects with other conditions that could cause LV hypertrophy (LVH) or myocardial disease, such as ischemic heart disease (IHD), valvular heart disease and cor pulmonale were all excluded. IHD was defined by the presence of any of the following: history of angina or IHD, electrocardiographic changes suggestive of myocardial infarction, and regional wall motion abnormalities on echocardiography. None of the subjects had a history of any form of cardiac surgery.

Hypertension was defined as systolic blood pressure (SBP) ≥ 140 mmHg and/or diastolic blood pressure (DBP) ≥ 90 mmHg, according to standard recommendations by the World Health Organisation.^15^ Hypertensive LV geometric patterns were defined as above and illustrated in Fig. 1.^5^ RWT was calculated using the following formula: RWT = $\frac{2\left(LV\;posterior\;wall\;thickness\;at\;end-diastole\right)\;(in\;mm)\;}{LV\;\;end-diastolic\;dimension\;(in\;mm)\;}$.^2^

Normal RWT was defined as values ≤ 0.42, and was increased if RWT was > 0.42. Increased LV mass index (LVMI) was defined as values > 125 g/m^2^ for all subjects.^5^ Proximal RV outflow tract dimension at end-diastole (RVOTd) was used as the measure for right ventricle size.^13^,^16^

RVSD was defined as either TAPSE of < 16 mm, or peak velocity of < 10cm/s of the systolic wave (S_m_) in tissue Doppler imaging (TDI) of the RV lateral tricuspid annulus, or both.^17^ RVDD was defined as the ratio of < 1.0 of peak velocities of the early (E_m_) to late (A_m_) diastolic waves in the TDI of the RV lateral tricuspid annulus, which was reported to represent global RV diastolic function.^2^

Pulmonary artery systolic pressure (PASP) was estimated using continuous-wave Doppler echocardiography, which was used to measure the maximum velocity of the tricuspid regurgitant jet (v), with which the trans-tricuspid pressure gradient was calculated using the modified Bernoulli equation (4v^2^).^18^ RV systolic pressure (RVSP) was then estimated by adding the trans-tricuspid pressure gradient to the right atrial pressure (RAP).^18^ RVSP was then equated to the PASP, given that pulmonary valve stenosis was excluded.^18^ RAP was then estimated using the diameter and collapse of the inferior vena cava (IVC) during spontaneous respiration, as previously described.^19^

Subjects in the NG group were used as controls to compare with the others who had abnormal LV geometric patterns.

Data were analysed with SPSS version 16.0. Means and standard deviations were computed and presented for quantitative variables. Student’s *t*-test, Fisher’s exact and Chi-square (χ^2^) tests were used for comparison between groups, as appropriate. Univariate regression and binary logistic regression models, and Pearson’s correlation (*r*) coefficient were used to analyse the associations between indices for RVSD and RVDD and a number of variables. Results for regression models were expressed in odds ratios (OR) and 95% confidence intervals (95% CI). A *p*-value < 0.05 was regarded as significant.

## Results

A total of 128 subjects were serially recruited, and the results for RV function and clinical characteristics are presented in Table 1. There were more females (69; 53.9%) than males (59; 46.1%) in the series. The mean age of all subjects was 51.04 ± 14.24 years, while the mean SBP was 154.91 ± 32.39 mmHg and mean DBP was 94.89 ± 18.51 mmHg. Overall, the prevalence of RVDD almost doubled that of RVSD in the studied subjects (61.72 vs 32.03%, respectively).

**Table 1.** Pattern Of RV Dysfunction And Characteristics Of Subjects Grouped According To LV Geometric Patterns

*Characteristics*	*CH (n = 29)*	*EH (n = 38)*	*CR (n = 7)*	*NG (n = 54)*
RVSD	6 (20.69)	20 (52.63)**	3 (42.86)	12 (22.22)
RVDD	16 (55.17)	23 (60.53)	3 (42.86)	37 (68.52)
TAPSE (mm)	19.35 ± 4.33	17.12 ± 4.92**	19.52 ± 4.47	20.48 ± 5.00
S_m_ (cm/s)	15.21 ± 4.63	12.61 ± 5.31**	15.28 ± 5.95	16.30 ± 5.56
E_m_:A_m_	0.92 ± 0.52	0.95 ± 0.59	1.11 ± 0.94	0.99 ± 0.64
RVOTd (mm)	28.62 ± 7.34	29.47 ± 5.83**	28.17 ± 3.60	26.08 ± 4.21
M/F	19/10**	21/17*	2/5	17/37
Age (years)	49.97 ± 13.34	52.95 ± 15.20	59.67 ± 22.21	49.35 ± 12.90
BMI (kg/m^2^)	25.01 ± 3.88**	25.18 ± 4.15**	22.22 ± 3.77*	30.14 ± 6.88
SBP (mmHg)	173.57 ± 35.00	136.31 ± 26.48	160.35 ± 21.05	153.56 ± 28.11
DBP (mmHg)	102.14 ± 21.19	83.08 ± 14.37*	100.62 ± 18.23	97.50 ± 16.11
Heart rate/min	89.55 ± 35.80	93.50 ± 14.36	82.82 ± 18.69	85.12 ± 16.96
Smoking	2 (6.9)	2 (5.3)	0	4 (7.4)
LA (mm)	39.90 ± 7.64**	42.03 ± 7.24**	40.14 ± 6.87*	34.33 ± 6.07
LVEDD (mm)	50.93 ± 4.78**	64.95 ± 8.87**	46.71 ± 13.46	46.85 ± 5.74
LVEF (%)	53.13 ± 14.55**	32.99 ± 15.76**	54.00 ± 17.61*	65.36 ± 10.10
MV E:A	1.54 ± 1.72	1.85 ± 1.56	0.68 ± 0.26	1.17 ± 1.02
PASP (mmHg)	34.59 ± 22.18**	47.47 ± 33.02**	22.93 ± 16.23	22.80 ± 14.43
LVMI (g/m^2^)	189.03 ± 4015**	192.16 ± 57.47**	114.57 ± 8.40**	83.91 ± 22.52

M/F, male/female; BMI, body mass index; SBP and DBP, systolic and diastolic blood pressures, respectively; LA, left atrium; LVEDD, LV end-diastolic dimension; LVEF, left ventricular ejection fraction; MV, mitral valve; E:A, ratio of early to late peak filling velocities; PASP, pulmonary artery systolic pressure; LVMI, LV mass index; RVOTd, RV outflow tract proximal dimension at end-diastole. *p-value statistically significant at < 0.05 level, for comparisons between subjects with NG and others. **p-value statistically significant at < 0.01 level, for comparisons between subjects with NG and others. All values are expressed as means ± standard deviations, or as numbers with percentages in parentheses.

The majority of subjects (54; 42.2%) had NG, while 38 of them (29.7%) had EH, 29 (22.7%) had CH, and seven (5.5%) had CR. Subjects with EH had the highest prevalence of RVSD, the largest cardiac chambers, lowest mean blood pressures, and highest mean LVMI and PASP. The prevalence of RVDD was high across the groups, but the differences between them were not statistically significant (*p* > 0.05).

The pattern of antihypertensive prescriptions is presented in Table 2, and was similar across the groups (*p* ≥ 0.05). Overall, only 38 subjects (29.7%) had controlled systolic (< 140 mmHg) and diastolic (< 90 mmHg) blood pressures at the time of recruitment into the study, and blood pressure was controlled in the majority of subjects with EH (55.3%).

**Table 2.** Pattern Of Antihypertensive Prescriptions Among All Subjects

*Type of antihypertensive*	*EH 38 (29.7)*	*CH 29 (22.7)*	*CR 7 (5.5)*	*NG 54 (42.2)*
CCB	8 (21.1)	5 (17.2)	3 (42.9)	8 (14.8)
Thiazides	7 (18.4)	7 (24.1)	2 (28.6)	12 (22.2)
ACEI/ARB	5 (13.2)	4 (13.8)	0	4 (7.4)
Combinations/others	18 (47.4)	13 (44.8)	2 (28.6)	30 (55.6)

CCB, calcium channel blockers; ACEI, angiotensin converting enzyme inhibitors, ARB, angiotensin II receptor blockers. All values are expressed as numbers with percentages in parentheses.

Univariate regression analyses were carried out to test for variables associated with RVSD or RVDD. Age was found to be the only variable that was associated with RVDD, with an OR of 1.032, 95% CI of 1.004–1.060, and *p*-value of 0.023. Several variables were found to be significantly (*p* < 0.05) associated with RVSD in univariate analyses. These variables were left atrial dimension, LV end-diastolic dimension (LVEDD) and volume (LVEDV), LV end-systolic dimension (LVESD) and volume (LVESV), LVEF and PASP. However, LVEF was the only variable that was independently associated with RVSD after controlling for the confounding factors (OR = 0.943; 95% CI = 0.897–0.993; *p* = 0.025).

The correlates for the indices of RVSD and RVDD are presented in Table 3. The only correlate for the Em:Am ratio was age (*r* = –0.237, *p* = 0.016), while several variables were found to correlate with both TAPSE and S_m_.

**Table 3.** Correlates Of Indices Of RVSD And RVDD In All Subjects

*Variables*	*TAPSE (mm)*	*S_m_ (cm/s)*	*E_m_:A_m_*
Age (years)			
*r*	–0.089	< 0.081	–0.237
p*-value*	0.351	0.398	0.016*
BMI (kg/m^2^)			
*r*	< 0.152	< 0.081	+0.000
p*-value*	0.183	0.487	0.999
RVOTd (mm)			
*r*	–0.126	–0.166	< 0.320
p*-value*	0.204	0.103	0.746
LVEDD (mm)			
*r*	–0.230	–0.299	< 0.320
p*-value*	0.014*	0.001*	0.746
LVEF (%)			
*r*	< 0.410	< 0.360	–0.064
p*-value*	< 0.001*	< 0.001*	0.519
LVMI(g/m^2^)			
*r*	–0.189	–0.146	–0.070
p*-value*	0.045*	0.122	0.478
MV E:A ratio			
*r*	< 0.005	–0.173	< 0.064
*p-value*	0.957	0.073	0.521
PASP (mmHg)			
*r*	–0.353	–0.190	< 0.147
p*-value*	< 0.001*	0.059	0.152

All values are expressed as means ± standard deviations or as numbers with percentages in parentheses; *p-value is statistically significant.

In univariate analyses, the main determinants of having NG in the present series were higher BMI and LVEF, and reduced RVOTd, left atrial diameter, LVEDD, LVESD and PASP (*p* < 0.01 for each). However, BMI (OR = 2.011; 95% CI = 1.100–3.679; *p* = 0.023) and LVEF (OR = 1.273; 95% CI = 1.042–1.555; *p* = 0.018) were the only determinants of NG that maintained their significance after controlling for other factors.

## Discussion

This study describes the pattern of RVSD and RVDD in a hypertensive population grouped by LV geometric patterns. The overall prevalence of RVDD was higher than that of RVSD, and the highest prevalence of the latter was recorded in subjects with EH, while prevalence of RVDD was high across the groups. The study has also described the determinants and correlates of RVSD and RVDD.

Several studies have previously reported that RV disease develops in parallel with a similar process on the left side among hypertensive patients,^1^,^2^ likely as a result of ventricular interdependence. Ventricular interdependence is defined as the forces that are transmitted directly from one ventricle to the other through the myocardium and pericardium, independent of neural, humoral or circulatory effects. It is a consequence of the close anatomical association between the ventricles: the ventricles are encircled by common muscle fibres, share a septal wall, and are enclosed within the pericardium.^20^

In agreement with this hypothesis, LVEF, the index for LV systolic function, was the only independent determinant of RVSD. In addition, the present study also recorded the highest prevalence of RVSD among subjects with EH who had the lowest mean LVEF and the worst LV systolic function, while the lowest prevalence of RVSD was recorded among those with NG, who correspondingly had the highest mean LVEF.

The prevalence of RVSD in the present study (32.03%), determined using both TAPSE and S_m_, was only slightly higher than what we reported previously (29.06%) using TAPSE alone.^1^ This suggests that the inclusion of S_m_ in the determination of RVSD contributes very little over that of using TAPSE alone. This finding therefore supports the use of TAPSE alone to determine RVSD in hypertensive subjects.

The prevalence of RVSD in the present study was lower than what was reported by Puwanant *et al.* (58%) among hypertensive subjects using reduced TAPSE (< 15 mm) alone, perhaps because 51% of the patients in their series had coronary artery disease, 37% had diabetes mellitus and 32.5% had cardiomyopathies.^21^ In addition, the patients in the latter study were older than ours (mean age 72 ± 14 vs 51 ± 14 years), and we have previously shown that older age is significantly associated with reduced TAPSE.^1^ Furthermore, differences in the aetiology of heart disease in the two studies could have amplified the disparities in RVSD.^21^

The prevalence of RVDD was high across the LV geometric groups, without significant statistical differences. Therefore, the pattern of RVDD in the studied population was not similar to that of RVSD. In the present study, subjects with NG, who had a mean mitral filling E:A ratio of 1.17 and the lowest mean age and RVOTd, turned out to have (albeit non-significantly) the highest prevalence of RVDD. We found that age was the only determinant of RVDD (*p* = 0.023), and correlated significantly and negatively with the lateral tricuspid annular E_m_:A_m_ ratio; the index of RVDD (*r* = –0.237; *p* = 0.016). The relatively lower mean age of patients with NG could therefore have influenced the observed high prevalence of RVDD in them, in view of the negative correlation between age and the E_m_:A_m_ ratio.

Innelli *et al*. recently reported that the E_m_:A_m_ ratio consistently and progressively decreased with age in an apparently healthy population, from 1.9 ± 0.8 in the 10–19-year-olds to 0.95 ± 0.3 in 50–59-year-old age group.^22^ It then appears that an Em:Am ratio of < 1.0 may not be reliable for the assessment of RVDD in a young population, such as those in the NG group. The dependence of RVDD on age could be attributed to the increase in arterial stiffness of the pulmonary vessels with ageing.^23^

Another possible explanation for the high prevalence of RVDD across the groups in our study could be the fact that RVDD in hypertensives has been shown to develop early, before apparent systolic dysfunction, and before RV dilatation or RV hypertrophy.^17^ This implies that RVDD could be the earliest index of RV affectation in hypertension, and perhaps occurs before LV geometry becomes abnormal. Therefore, the E_m_:A_m_ ratio could potentially be used as a high-sensitivity screening tool for RV disease, but taking the age of the individual into consideration.

In contrast to our finding, Cicala *et al.* previously reported that mitral annular E_m_:A_m_ ratio and body mass index were the only predictors of RVDD, while age, DBP, heart rate, septal and RV wall thickness were not associated with the RVDD.^2^ We found no relationship between RVDD and LVMI, but did not assess the relationship between RVDD and mitral annular or mitral valve filling variables in the present study.

Several conditions have been associated with RVDD, including both RV pressure and volume overload pathologies, primary lung disease, IHD, congenital heart disease, cardiomyopathies, LV dysfunction (via ventricular interdependence), systemic diseases and the physiological aging process.^17^ IHD is still uncommon in sub-Saharan Africa, previously found among 8.7% of subjects referred for echocardiography in Kano, Nigeria, and among 10.4% of the Soweto community in South Africa.^9^,^24^ We excluded subjects with IHD, however, from the present study.

The limitations of the present study include the use of E_m_:A_m_ ratio alone to assess RVDD. However, this index is one of the recommended indices approved by the American Society of Echocardiography for assessing RVDD, and has been shown to represent global RV diastolic function.^2^,^17^ Secondly, magnetic resonance imaging appears to be superior to echocardiography and other techniques in studying the right ventricle.^25^ However, echocardiography has acceptable sensitivity, and is widely available and affordable, and therefore has an important role in studying the right ventricle, despite its limitations.

Another limitation was the use of Teicholz’s formula to estimate LVEF, which has the inherent tendency to over-estimate it in the presence of abnormal LV geometry.^14^ To minimise the inaccuracy of the Teichholz’s formula, subjects with regional wall motion abnormality were excluded from the study. The formulae that estimate LV mass using measurements obtained from two-dimensional guided M-mode echocardiography have several limitations, including sub-optimal accuracy in the presence of abnormal LV geometry, large inter-observer variability and poor inter-study reproducibility.^14^,^16^ To minimise this, about 50% of the echocardiograms were carried out by the principal author (KMK), while the remaining 50% were carried out by the co-authors (HS and MNS). We are presently in the process of estimating the inter-observer variability for our echocardiography laboratory.

## Conclusion

This study has described, perhaps for the first time, the pattern of RVSD and RVDD in hypertensive subjects grouped according to pattern of LV geometry. The majority of subjects (42.2%) had NG, while 29.7% had EH, 22.7% had CH, and 5.5% had CR. The highest prevalence of RVSD was recorded in subjects with EH who had the lowest mean LVEF and the worst LV systolic function, while the prevalence of RVDD was high across the groups.

LVEF was the only independent determinant of RVSD after controlling for confounding variables, while age was the only determinant of RVDD. Likewise, age was the only correlate for E_m_:A_m_ ratio, while the best correlate for both TAPSE and S_m_ was LVEF. These results have further characterised both LV and RV geometry and function in subjects with hypertension.
